# Supplementary material for: Oceanic memory of tropical cyclones moderates the Kuroshio current
Source: Nat Commun. 2025 Jul 26;16:6890. doi: 10.1038/s41467-025-62239-2 (PMC12297435; doi:10.1038/s41467-025-62239-2)
Supplement: Supplementary file 1 — Supplementary Information [file 41467_2025_62239_MOESM1_ESM.pdf]

Supplementary Information for

Oceanic memory of tropical cyclones modulates the Kuroshio current

Deyuan Zhang[1&], Zhanhong Ma[1&*], Lijing Cheng[2*], Yanluan Lin[3], Fanghua Xu[3], Zhengguang Zhang[4,5], Yunxia Zheng[6], Jianfang Fei[1], and Michael E. Mann[7,8]

[1] *College of Meteorology and Oceanography, National University of Defense Technology, Changsha, China*

[2] *State Key Laboratory of Earth System Numerical Modeling and Application, Institute of Atmospheric Physics, Chinese Academy of Sciences, Beijing, China*

[3] *Department of Earth System Science, Ministry of Education Key Laboratory for Earth System Modeling, Institute for Global Change Studies, Tsinghua University, Beijing, China*

[4] *Frontiers Science Center for Deep Ocean Multispheres and Earth System (FDOMES) and Key Laboratory of Physical Oceanography, Academy of the Future Ocean, Chongben Honors College, Ocean University of China, Qingdao, China*

[5] *Laoshan Laboratory, Qingdao, China*

[6] *Shanghai Typhoon Institute, China Meteorological Administration, and Key Laboratory of Numerical Modeling for Tropical Cyclone, China Meteorological Administration, Shanghai, China*

[7] *Department of Meteorology, Pennsylvania State University, University Park, Pennsylvania, USA*

[8] *Earth and Environmental Systems Institute, Pennsylvania State University, University Park, Pennsylvania, USA*

Contents of the file

Supplementary Fig.1 to Fig. 16

*Corresponding to: Zhanhong Ma (mazhanhong17@nudt.edu.cn);

Lijing Cheng (chenglij@mail.iap.ac.cn)

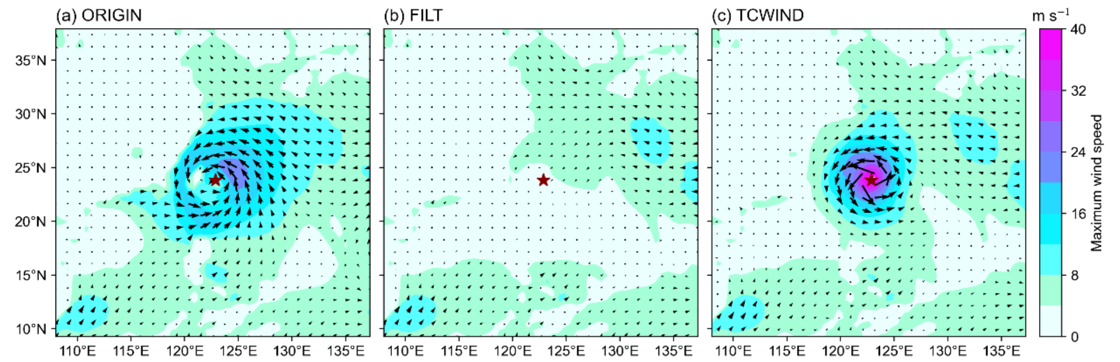

Supplementary Fig. 1. Example for the reconstruction of tropical cyclones' (TCs) wind fields. Wind speed (shading) and direction (arrows) in (a) JRA55, (b) FILT (with TC removed), and (c) TCWIND (with TC embedded) experiments for super Typhoon Talim. The red star marks the position on 31 August 2005.

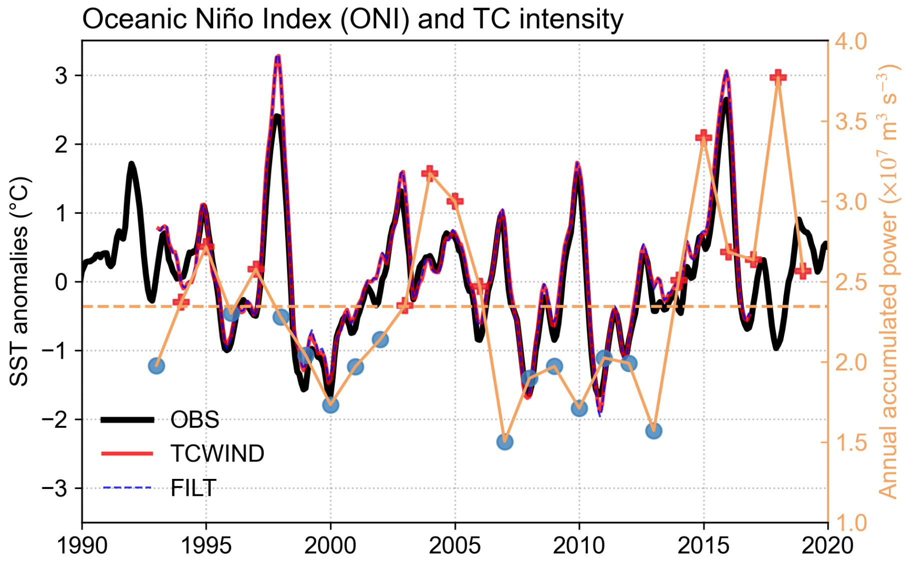

Supplementary Fig. 2. Time series of Oceanic Niño Index (ONI) and tropical cyclones' (TCs) intensity in the Kuroshio region. Oceanic Niño Index (ONI) is defined as a 3-month running mean of sea surface temperature anomalies in the Niño 3.4 region (5°S-5°N, 120°-170°W). TC intensity, defined as annual accumulated wind power input near the Kuroshio area (110° to 140°E, 15° to 35°N), is calculated at 1-

hour intervals using linearly interpolated position and maximum wind speed from the 6-hourly IBTrACs best-track archive. The dashed yellow line shows the average intensity from 1993 to 2019. The TC-rich and TC-poor years are marked by red plus and blue circles, respectively.

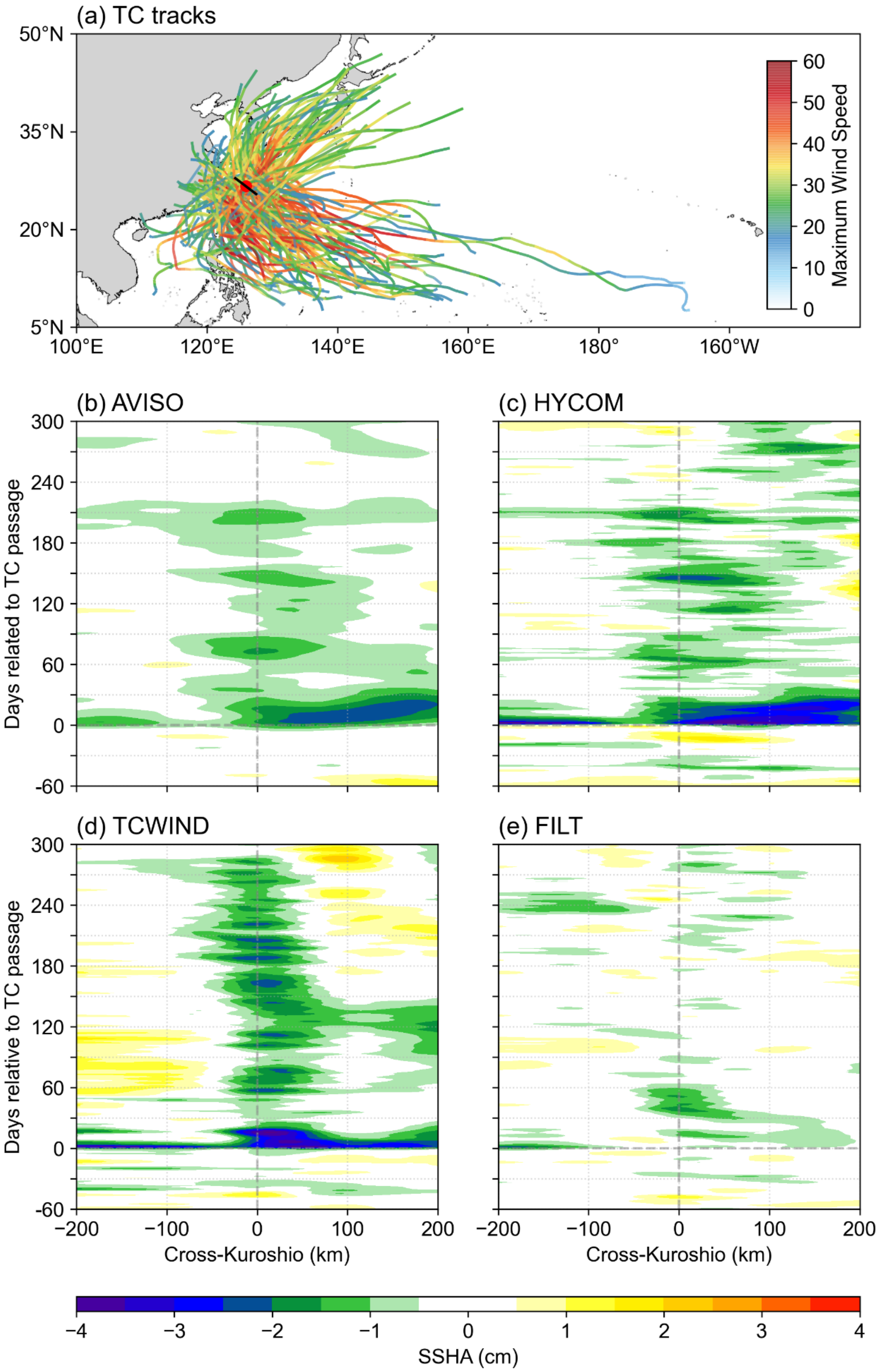

Supplementary Fig. 3. Temporal evolution of composite sea surface height

anomaly (SSHA; cm) at section 7 associated with the passage of tropical cyclones (TCs). (a) The tracks and intensity (maximum wind speed) of TCs within 500 km of the section center. Temporal evolution of SSHA at section 7 before and after the TC passage from (b) AVISO, (c) HYCOM, (d) the TCWIND experiment, and (e) the FILT experiment. The linear trend and seasonal cycle have been removed.

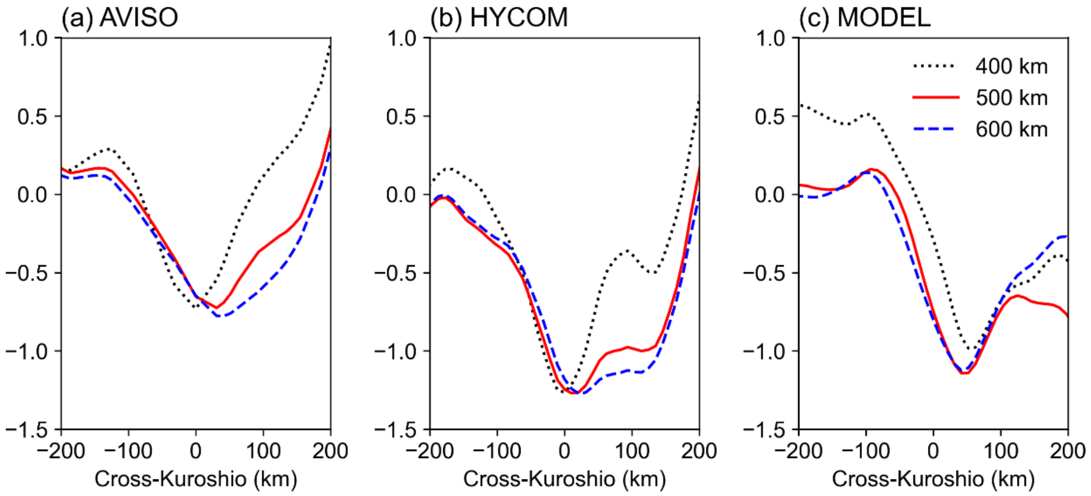

Supplementary Fig. 4. Composite sea surface height (SSH) changes across section 7 after the passage of tropical cyclones (TCs) with different distance thresholds. The first time when TC position is within 400 km (black dotted lines), 500 km (red solid lines), and 600 km (blue dashed lines) is defined as day 0 when a TC starts to impact the section. The SSH anomaly (cm) is calculated as the averaged value over a period of 120 to 180 days after TC passage relative to the pre-storm state (days -30 to -3) derived from (a) AVISO, (b) HYCOM reanalysis, and (c) the TCWIND experiment. The seasonal cycle and linear trend have been removed.

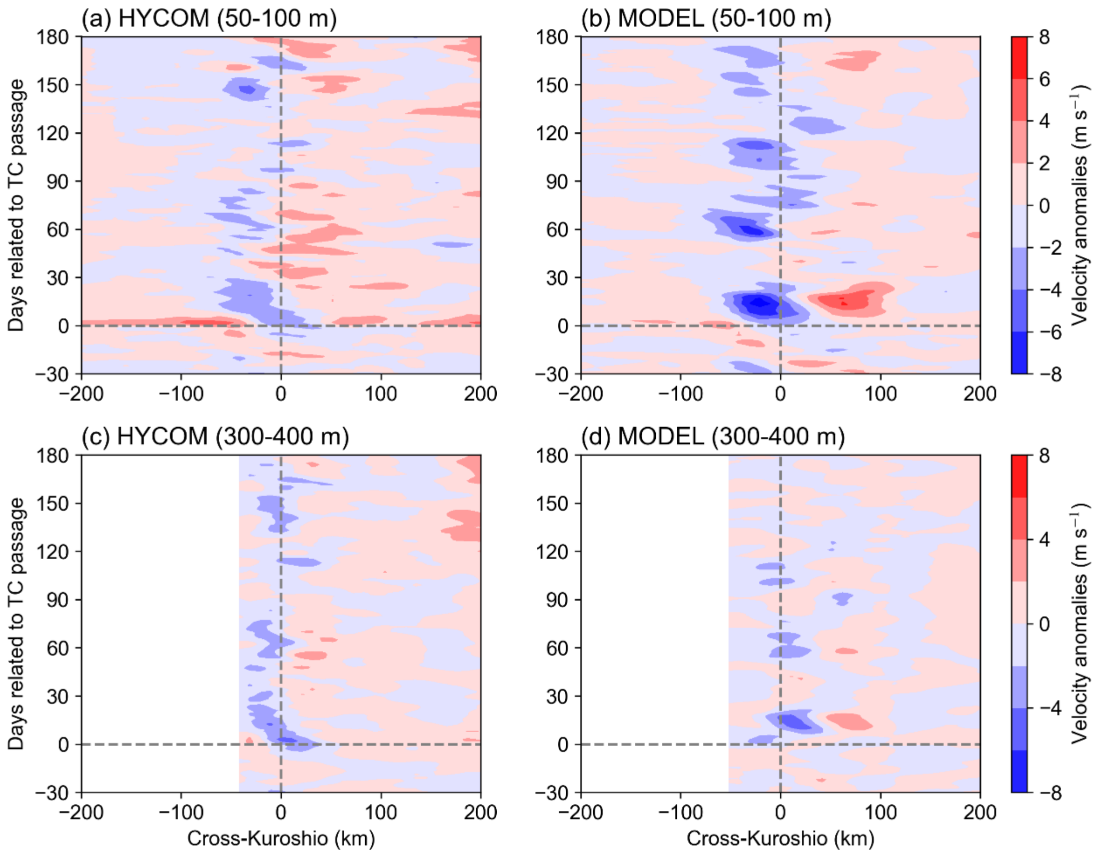

Supplementary Fig. 5. Temporal evolution of composite velocity anomalies at section 7 associated with the passage of tropical cyclones (TCs). Temporal evolution of averaged velocity across section 7 over (a, b) 50-100 m and (c, d) 300-400 m derived from (a, c) the HYCOM reanalysis and (b, d) the TCWIND experiment (with TC embedded). The linear trend and seasonal cycle have been removed.

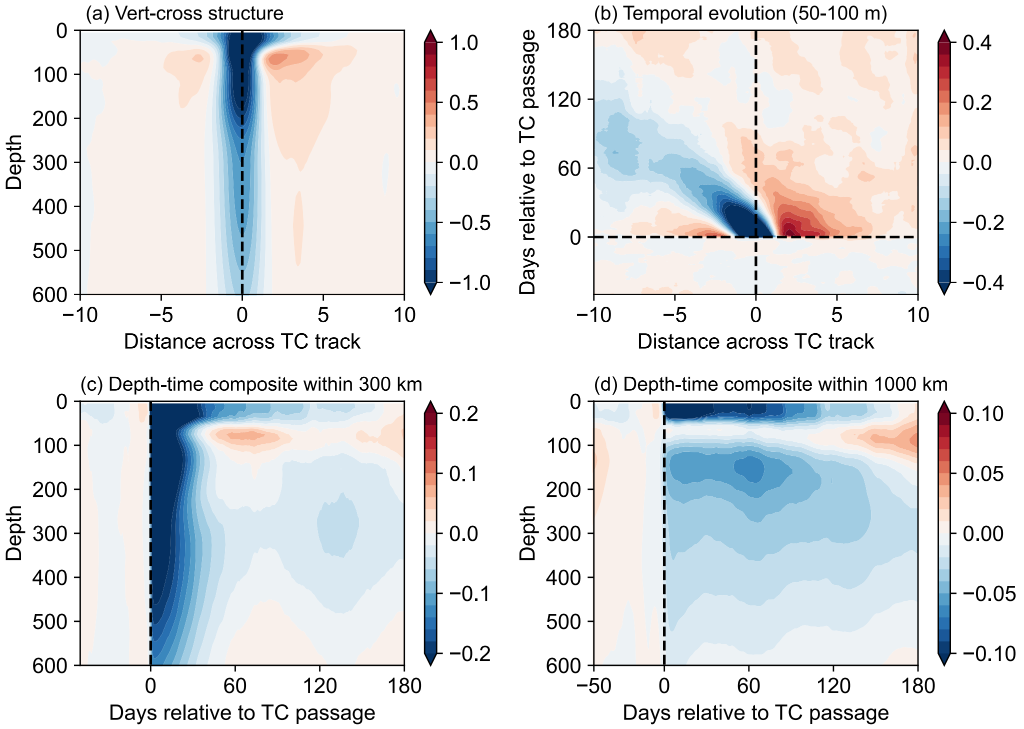

Supplementary Fig. 6. Composite potential temperature anomalies induced by tropical cyclones (TCs) in the TCWIND experiment (with TC embedded). (a) Vertical profiles and (b) temporal evolution of along-track-averaged temperature anomalies. The along-track average is performed between -1° and 1° surrounding the TC center. The vertical section is averaged over day +3 to+5 after TC passage, while the temporal evolution over 50 to 100 m depth range. Depth-time composite of temperature anomalies within (c) 300 km and (d) 1000 km of the TC center. All composites encompass all TCs of category 1 to 5 intensity in the North Western Pacific (100° to 180°E; 0 to 40°N) during 1993-2016. The anomalies are calculated relative to the pre-TC passage period (days -30 to -3) with the seasonal cycle removed.

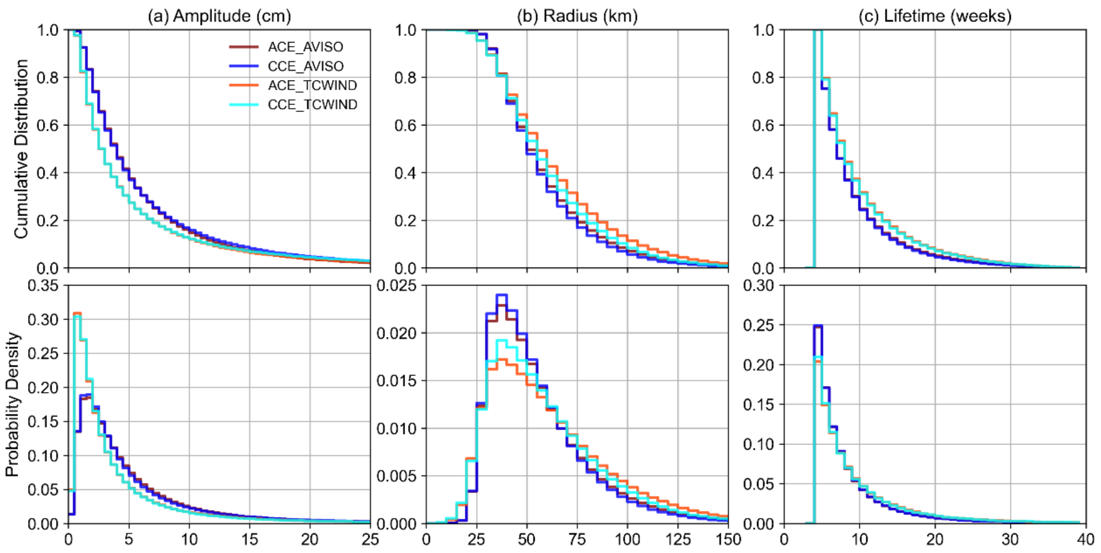

Supplementary Fig. 7. Verification of oceanic mesoscale eddies properties. Upper-tail cumulative histogram (first row) and histogram (second row) for (a) amplitude (cm), (b) radius (km), and (c) lifetime (weeks) during 1993-2016. The blue and brown curves represent the results for observed cyclonic and anticyclonic eddies (CCEs and ACEs) based on AVISO, respectively. The orange and cyan curves represent CCEs and ACEs based on the TC experiment (with TC embedded), respectively.

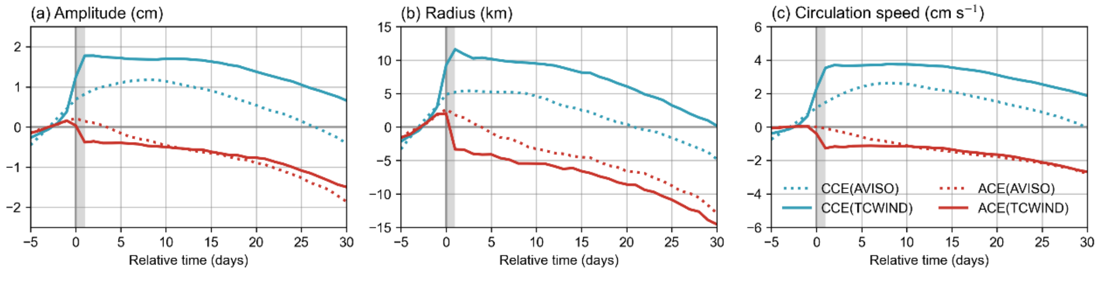

Supplementary Fig. 8. Changes in structural characteristics of oceanic mesoscale eddies induced by tropical cyclones (TCs) from observation and simulation. Temporal evolution of TC-induced changes in (a) amplitude (cm), (b) radius (km), and (c) circulation speed (cm s^{-1}) derived from AVISO (dashed lines) and the TC experiment (solid line) during 1993-2016. The TC and eddy start to interact when their central

distance is equal to or smaller than the eddy radius. The changes are calculated by subtracting the pre-storm state, which is time-averaged between day -5 and day -1. The red and blue curves represent anticyclonic eddies (ACE) and cyclonic eddies (CCE), respectively.

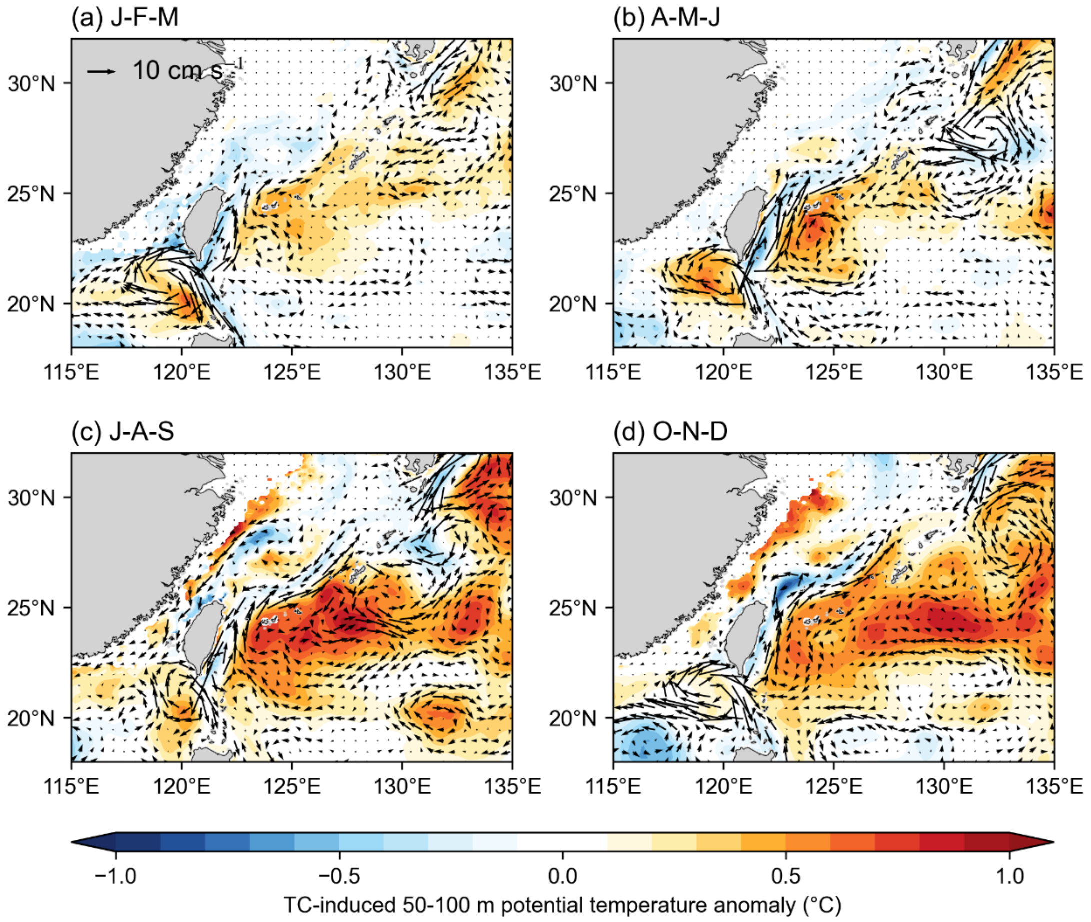

Supplementary Fig. 9. Simulated seasonal cycle of potential temperature anomalies (50-100 m; shading) and current velocity anomalies (0-50 m; vectors) induced by tropical cyclones (TCs). Panels show anomalies for (a) January to March (J-F-M), (b) April to June (A-M-J), (c) July to September (J-A-S), and (d) October to December (O-N-D). The anomalies are calculated as the difference of the long-term mean climatology (1993-2016) between the TCWIND (with TC embedded) and FILT

(with TC removed) experiments for respective seasons.

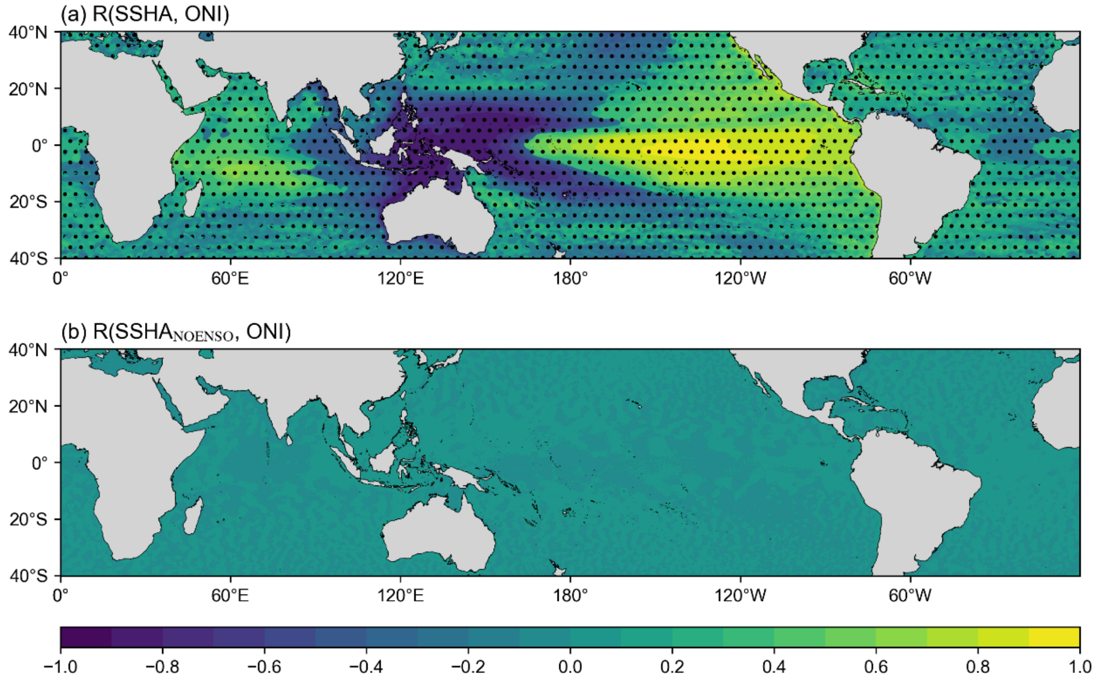

Supplementary Fig. 10. Verification of removing linear effect of El Niño–Southern Oscillation (ENSO). Maximum correlation coefficients between Oceanic Niño Index (ONI) and (a) sea surface height anomaly (SSHA) and (b) SSHA without the effects of ENSO within leads and lags of up to 12 months. SSHA is obtained by removing the seasonal cycle and linear trend. Stippled regions indicate that the correlation is significant at the 95% level.

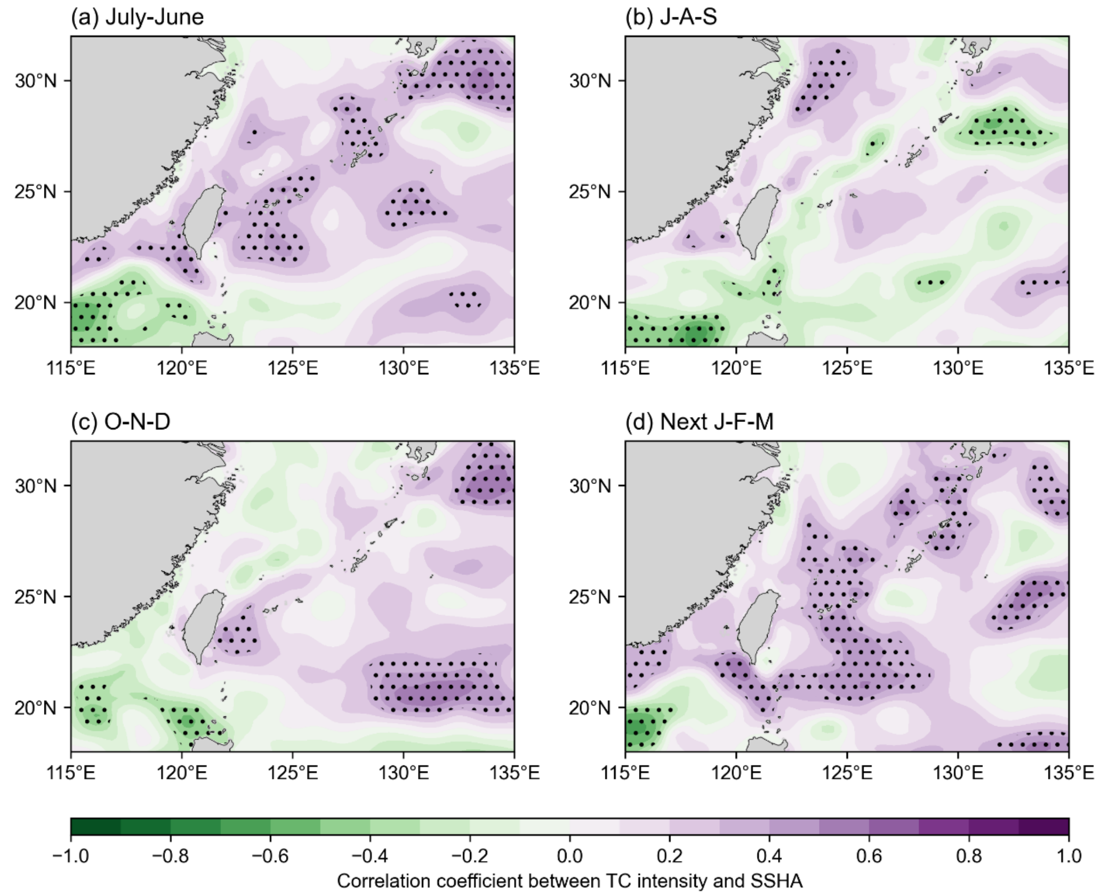

Supplementary Fig. 11. Correlation between the intensity of tropical cyclones (TCs) and sea surface height anomaly (SSHA). Spatial distribution of correlation coefficients between yearly TC intensity and SSHA averaged over (a) July–June, (b) July–August–September (J-A-S), (c) October–November–December (O-N-D), and (d) January–February–March (J-F-M) of the following year. Seasonal cycles, linear trends, and linear lead–lag influences (up to 12 months) associated with El Niño–Southern Oscillation (ENSO) have been removed to obtain SSHA. Stippling denotes correlations significant at the 90% confidence level.

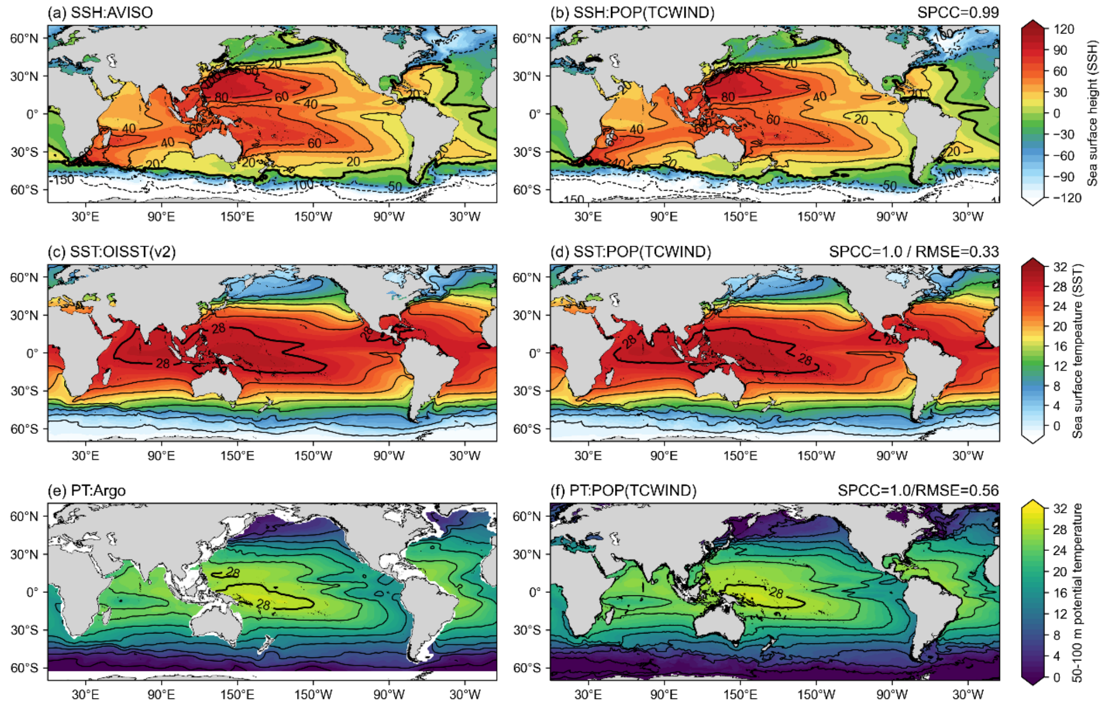

Supplementary Fig. 12. Verification of large-scale ocean climatology. Mean sea surface height (SSH, cm) of (a) AVISO and (b) the TCWIND experiment (with TC embedded) simulation during 1993-2016. Mean sea surface temperature (SST, °C) of (c) OISSTv2 and (d) the TCWIND experiment during 1993-2016. The mean subsurface temperature averaged over 50 to 100 m of (e) IPRC gridded Argo products and (f) the TCWIND experiment from 2005 to 2016. The spatial pattern correlation coefficient (SPCC) and root-mean-square error (RMSE) between the observation and simulation are noted on the top right panel of each variable.

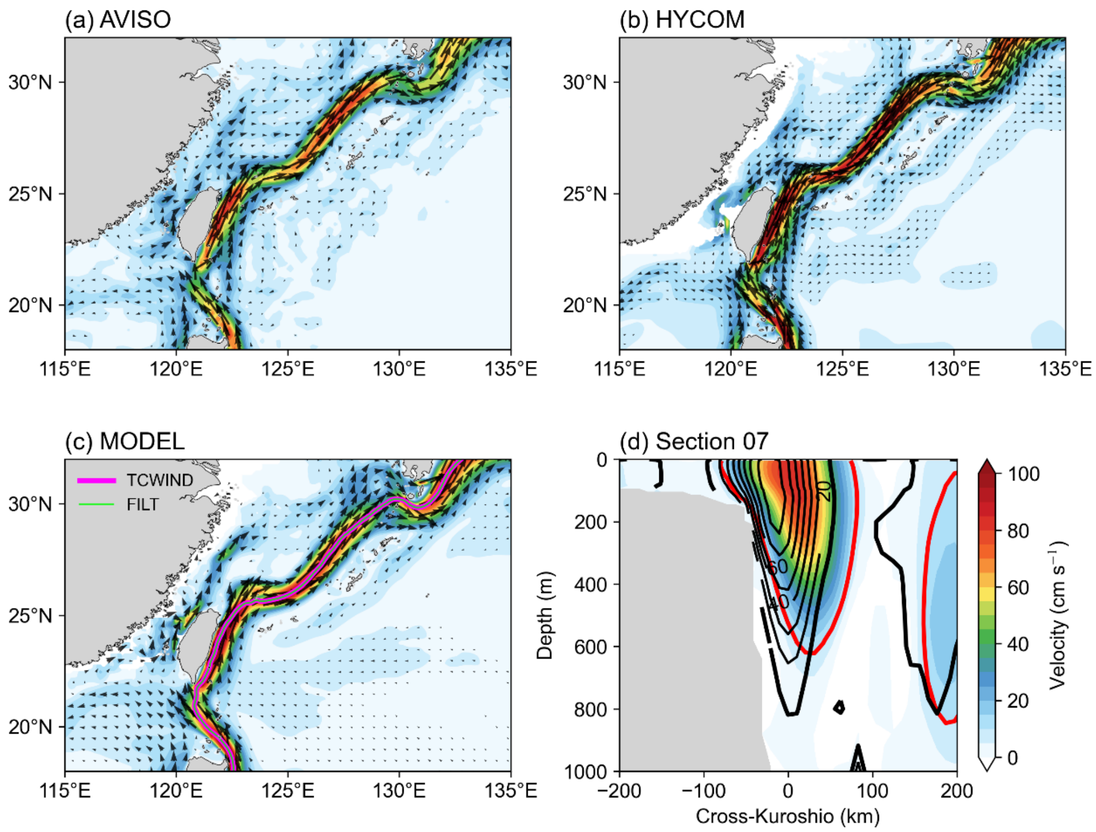

Supplementary Fig. 13. The climatology of the Kuroshio current from 1993 to 2016.

(a) The surface geostrophic velocity is derived from AVISO. The averaged velocity with upper 50 m from (b) the HYCOM reanalysis and (c) the TCWIND (with TC embedded) experiment. The solid lines designate the main axis of the Kuroshio based on velocity field. (d) The vertical structure of section 7 indicated in Fig. 1. The color shading is derived from the TCWIND experiment with red contours denoting the 10 cm s^{-1} threshold. The black contours are from the HYCOM reanalysis at an interval of 10 cm s^{-1}.

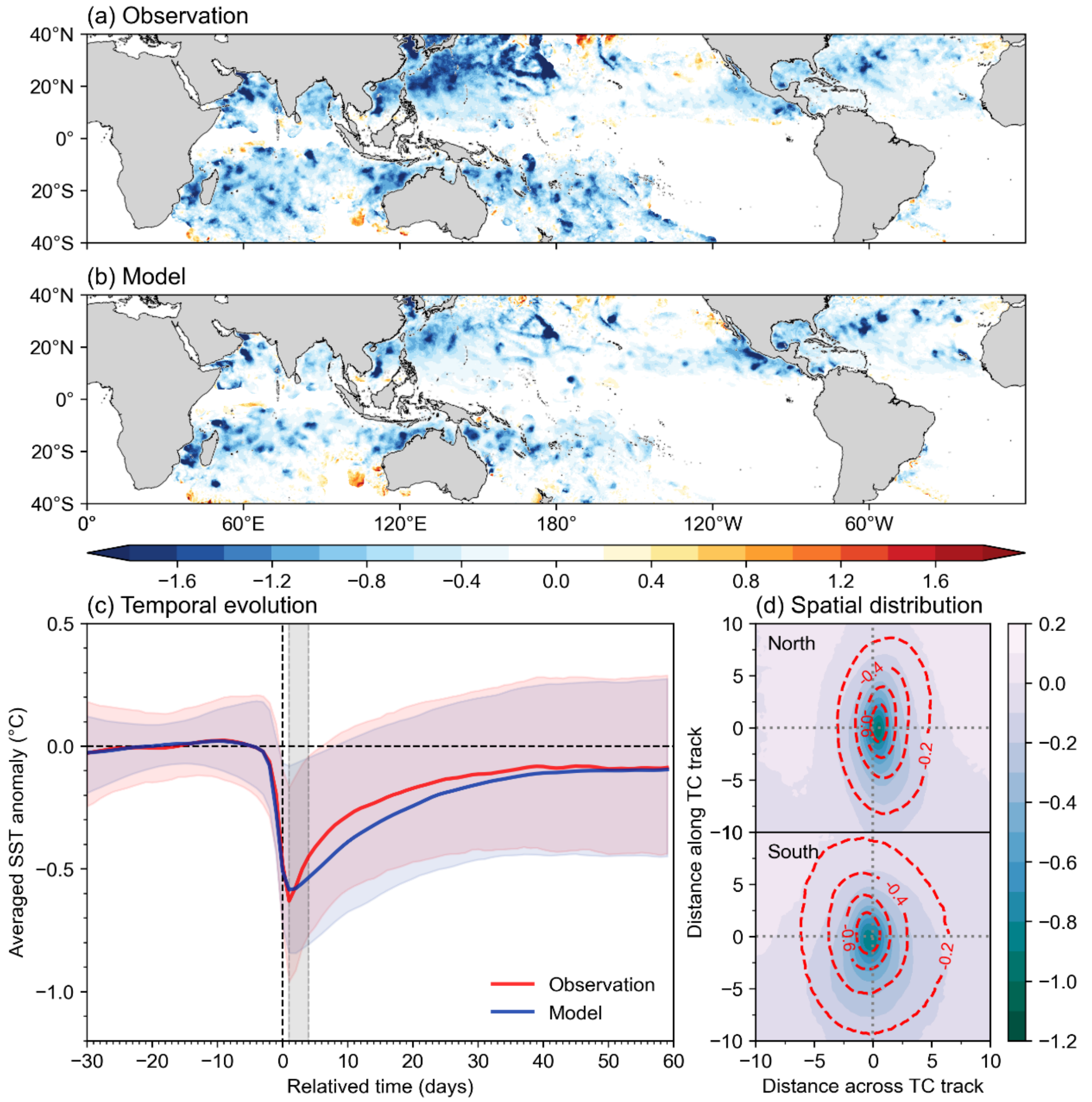

Supplementary Fig. 14. The cooling of sea surface temperature (SST) induced by tropical cyclones (TCs) from observation and simulation. Spatial distribution of averaged SST anomalies from day 1 to day 3 over 2002-2016 for (a) observation (high-resolution MW_IR SST) and (b) the TCWIND experiment (with TC embedded). Note that the SST anomalies within 200 km of each TC position are counted. (c) Composite temporal evolution of averaged SST anomalies (°C). The average area is within a 200 km radius of the TC center. Shading indicates the spread around the mean value, evaluated from the lower and upper quartiles. (d) Spatial distribution of SST anomalies

from day 1 to day 3 for TCs in the Northern Hemisphere and the Southern Hemisphere. The red contours are derived from observations while the color shading represents results in the TCWIND experiment. The SST anomalies are computed with respect to pre-storm SST (day-30 to day -3) with the seasonal cycle removed.

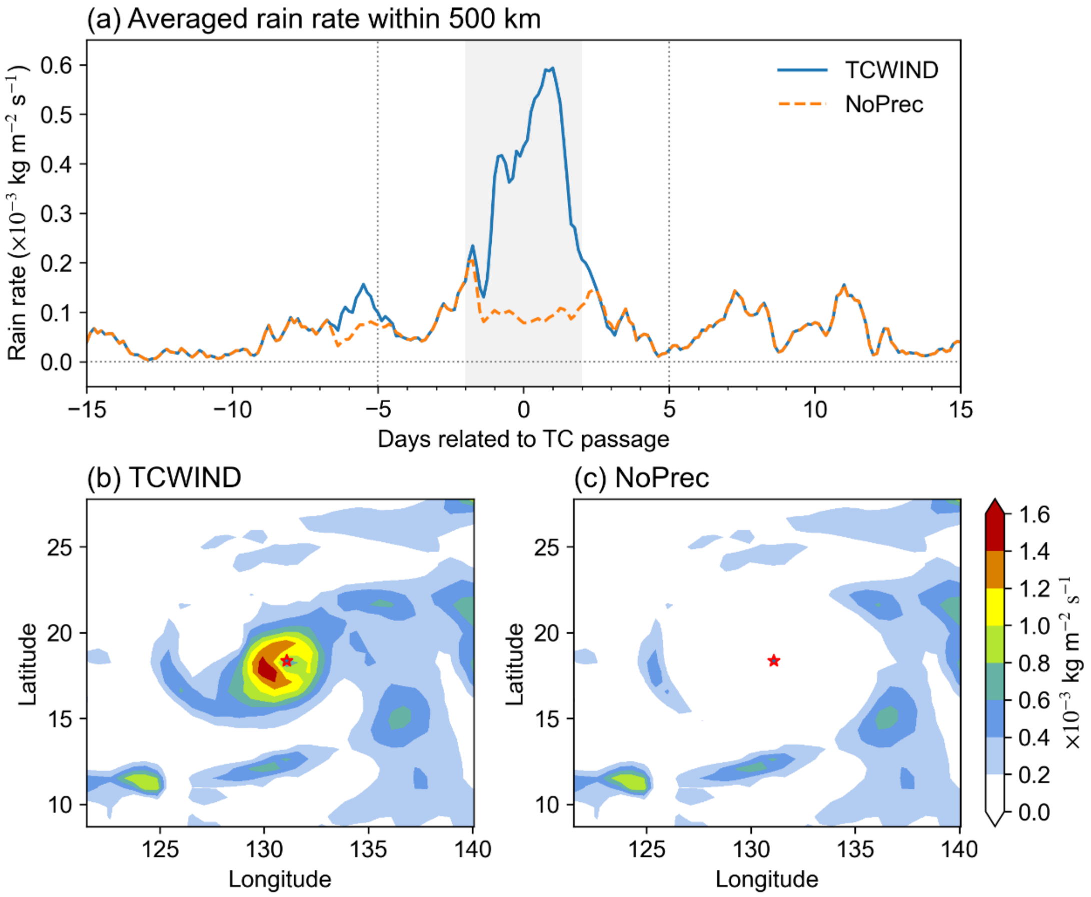

Supplementary Fig. 15. Example for removing precipitation related to tropical cyclones (TCs). (a) Time series of the averaged rain rate within 500 km. Spatial distribution of rain rate in (b) the TCWIND experiment (with TC's wind field embedded) and (c) the NoPrec experiment (with TC's rainfall removed) on day 0 (2014-10-8T10:30:00Z). The red star marks the position of TC Super Typhoon Vongfong.

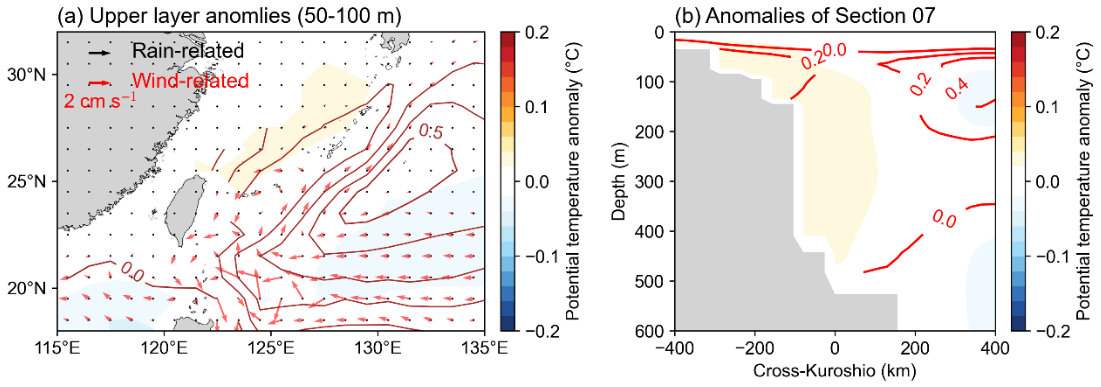

Supplementary Fig. 16. The simulated oceanic responses to precipitation of tropical cyclones (TCs) at a nominal 1° resolution. (a) TC precipitation-induced anomalies of potential temperature (shading) and current velocity (black vectors) averaged over 50-100 m depth. Red contours and vectors represent anomalies related to TC winds. (b) Vertical structure of potential temperature anomalies related to TC precipitation (shading) and TC wind (contours) across section 7. The anomalies associated with TC precipitation are calculated as the mean state difference between the TCWIND experiment and the NoPrec experiment.
